# NF-κB Regulates Caspase-4 Expression and Sensitizes Neuroblastoma Cells to Fas-Induced Apoptosis

**DOI:** 10.1371/journal.pone.0117953

**Published:** 2015-02-19

**Authors:** Hai-Jie Yang, Mian Wang, Lei Wang, Bin-Feng Cheng, Xiao-Yu Lin, Zhi-Wei Feng

**Affiliations:** College of Life Science and Technology, Xinxiang Medical University, Xinxiang, China; Roswell Park Cancer Institute, UNITED STATES

## Abstract

Found in neurons and neuroblastoma cells, Fas-induced apoptosis and accompanied activation of NF-κB signaling were thought to be associated with neurodegenerative diseases. However, the detailed functions of NF-κB activation in Fas killing and the effect of NF-κB activation on its downstream events remain unclear. Here, we demonstrated that agonistic Fas antibody induces cell death in a dose-dependent way and NF-κB signaling is activated as well, in neuroblastoma cells SH-EP1. Unexpectedly, NF-κB activation was shown to be pro-apoptotic, as suggested by the reduction of Fas-induced cell death with either a dominant negative form of IκBα (DN-IκBα) or an IκB kinase-specific inhibitor. To our interest, when analyzing downstream events of NF-κB signaling, we found that DN-IκBα only suppressed the expression of caspase-4, but not other caspases. *Vice versa*, enhancement of NF-κB activity by p65 (*RelA*) overexpression increased the expression of caspase-4 at both mRNA and protein levels. More directly, results from dual luciferase reporter assay demonstrated the regulation of caspase-4 promoter activity by NF-κB. When caspase-4 activity was blocked by its dominant negative (DN) form, Fas-induced cell death was substantially reduced. Consistently, the cleavage of PARP and caspase-3 induced by Fas was also reduced. In contrast, the cleavage of caspase-8 remained unaffected in caspase-4 DN cells, although caspase-8 inhibitor could rescue Fas-induced cell death. Collectively, these data suggest that caspase-4 activity is required for Fas-induced cell apoptosis and caspase-4 may act upstream of PARP and caspase-3 and downstream of caspase-8. Overall, we demonstrate that NF-κB can mediate Fas-induced apoptosis through caspase-4 protease, indicating that caspase-4 is a new mediator of NF-κB pro-apoptotic pathway in neuroblastoma cells.

## Introduction

Fas, also known as CD95, is a type I member belonging to tumor necrosis factor receptor (TNFR) superfamily [1,2]. It transmits death signals in many cell types [3–5]. Fas receptor contains an intracellular death domain. Once binding to Fas ligand (FasL) or its agonistic antibody, Fas can recruit and activate caspase-8 via Fas-associated death domain adaptor protein (FADD), forming death-inducing signaling complex (DISC). Next, DISC transduces death signal to its downstream effectors, caspase-3 and poly (ADP-ribose) polymerase (PARP), which induce cell death irreversibly via either mitochondria-dependent or mitochondria-independent pathways [5–7].

The Fas and FasL are widely expressed in normal central nerve system (CNS) [8,9], although their main function was initially supposed to kill invading lymphocytes. Strikingly, accumulated data have shown that Fas is involved in neuronal apoptosis not only for neuroblastoma [10–12] and neuron damage [13,14], but also for the apoptosis of cerebral cortex neurons and embryonic motoneurons [15–17]. Meanwhile, elevated expression of Fas/FasL is frequently found in various neurological diseases [8], suggesting that Fas/FasL system may play roles in degenerative responses in the CNS.

Fas elicits a pleiotropic response through transcriptional factor activation of a wide variety of genes [3,18], and one of them is NF-κB. The prototypical NF-κB is sequestered in the cytoplasm as inactive form bound by its endogenous inhibitors, inhibitory κB (IκB) family members. Upon Fas stimulation, NF-κB is activated together with its nuclear translocation. Blockage of NF-κB signaling sensitizes many cells to Fas-induced apoptosis, implying that NF-κB is required for transcription of genes beneficial for cell survival [8,18,19]. However, contrary reports have pointed out that the activation of NF-κB can promote apoptosis in other circumstances [20], e.g. in neurotoxin-induced neuron death [21], p53-dependent apoptosis [22], and Smac-induced apoptosis [23]. Notably, a pro-apoptotic role of NF-κB activation was observed in Fas-induced apoptosis in glioblastoma cells and endothelial cells [24,25]. Therefore, activation of NF-κB may confer either protective or apoptotic effect, depending on the stimuli utilized and the cell type. In cerebral cortex neurons and neuroblastoma cells, NF-κB was clearly influenced by Fas signal [8,16]. However, the function of NF-κB activation in Fas killing and the effect of NF-κB activation on its downstream events are largely unknown.

Here we show that NF-κB is activated during Fas-induced apoptosis in neuroblastoma cells SH-EP1, whereas the inhibition of NF-κB activity by dominant negative IκBɑ decreases sensitivity of neuroblastoma cells to Fas killing. Furthermore, we identified that NF-κB pro-apoptotic activity is mediated, at least in part, by caspase-4. Taken together, we reported here that NF-κB activation plays a pro-apoptotic role in Fas killing, and for the first time evidence was provided that NF-κB exerts its pro-apoptotic function through caspase-4.

## Results

### NF-κB is involved in Fas-induced cell death in neuroblastoma cells

**Fig 1:**
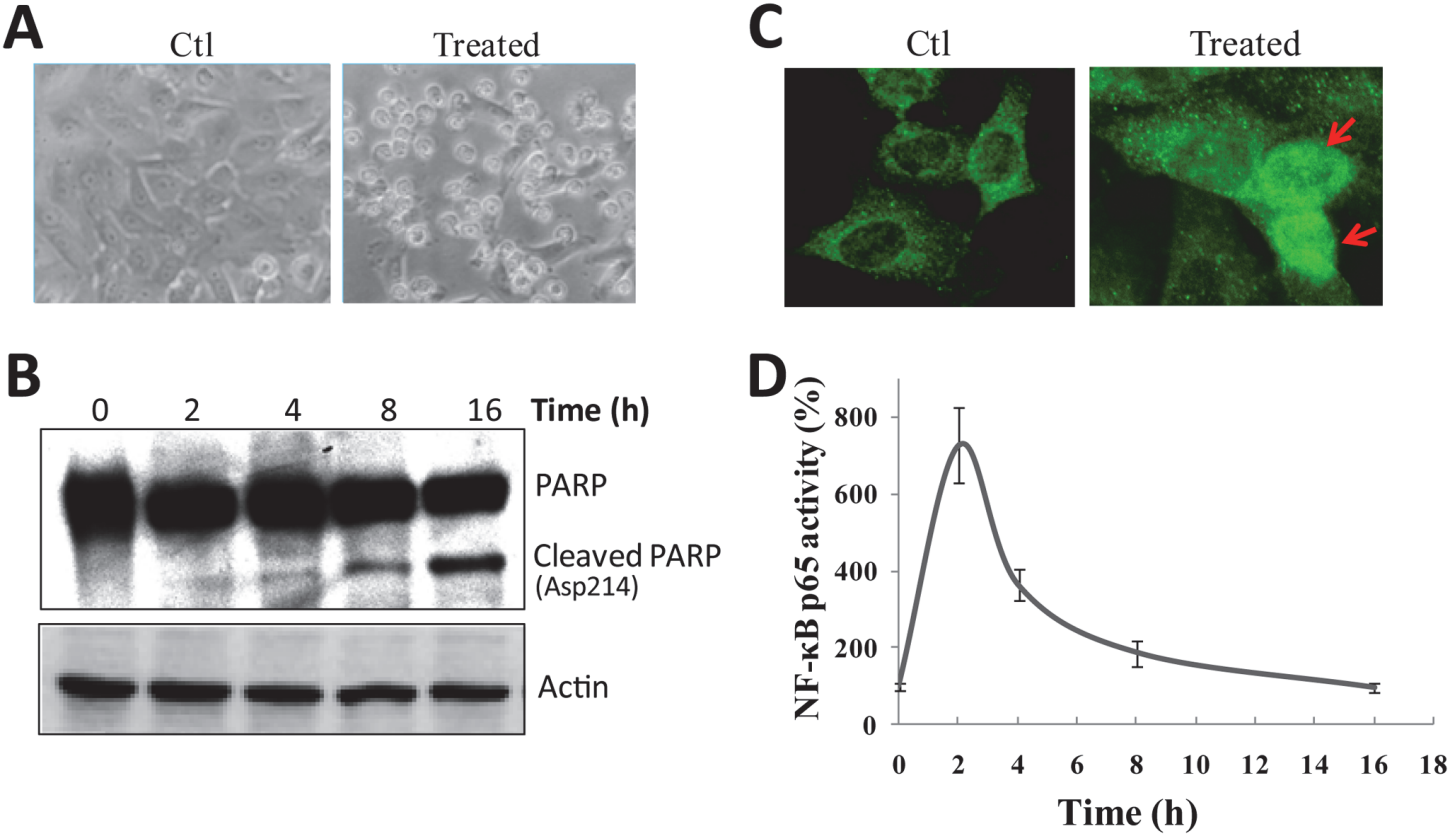
NF-κB is involved in Fas-induced cell apoptosis in neuroblastoma cells. (**A**) SH-EP1 cells were treated with agonistic anti-Fas antibody (100 ng/ml) for 1 d, and photographed live. Pictures are shown at ×100 magnification. (**B**) Immunocytochemistry targeting NF-κB p65 (green) was performed at 1 h after Fas treatment. Arrows indicate cells with p65 nuclear translocation. (**C**) Treated cells were lysed and Western blotting was performed with antibody against the cleaved PARP. Membranes were re-probed with β-actin as a loading control. Results are representative of at least three experiments. (**D**) After Fas treatment, NF-κB activation was analyzed using NF-κB p65 reporter assay. Data are from three repeated experiments and shown as average ± s.e.m. (bars) values. **P <0.01 and ***P <0.001, compared with untreated SH-EP1 cells.

Expression of both Fas and FasL in human neuroblastoma tissue and cell lines [11,26], provides an ideal model for investigating Fas/FasL in nervous system. To determine the role of NF-κB in Fas-induced apoptosis, we first examined whether Fas antibody induces apoptosis of SH-EP1 cells. As shown in Fig. 1A, when Fas antibody was added into cell culture medium, SH-EP1 cells exhibited apoptotic morphology, with cell body shrinking and membrane blebbing. Meanwhile, cleaved PARP fragment can be detected 4 h later (Fig. 1B), demonstrating that the cell death might start at around 4 h after Fas stimulation [27].

Previous report revealed the translocation of the main member of NF-κB, p65, to nucleus when stimulated by Fas in cerebral cortex neurons [17]. To clarify the involvement of NF-κB signaling in our system, we examined the nuclear translocation of p65 by immunocytochemistry. As shown in Fig. 1C, in untreated SH-EP1 cells, p65 was mainly sequestered in cytoplasm (left panel). In contrast, upon the addition of Fas antibody, a portion of p65 was translocated to nucleus (right panel).

To further confirm the activation of NF-κB by Fas stimulation, NF-κB p65 reporter assay was included. As shown in Fig. 1D, NF-κB activation was strongly induced by Fas antibody in SH-EP1 cells with a maximum activity at 2 h treatment. All together, these results clearly indicate the activation of NF-κB by Fas in SH-EP1 cells. The time course of NF-κB activation by Fas preceded the onset of apoptosis (about 4 h) after Fas treatment, suggesting that NF-κB activation may play a role in Fas-induced apoptosis.

### NF-κB inhibition protects neuroblastoma cells from Fas-induced cell death

**Fig 2:**
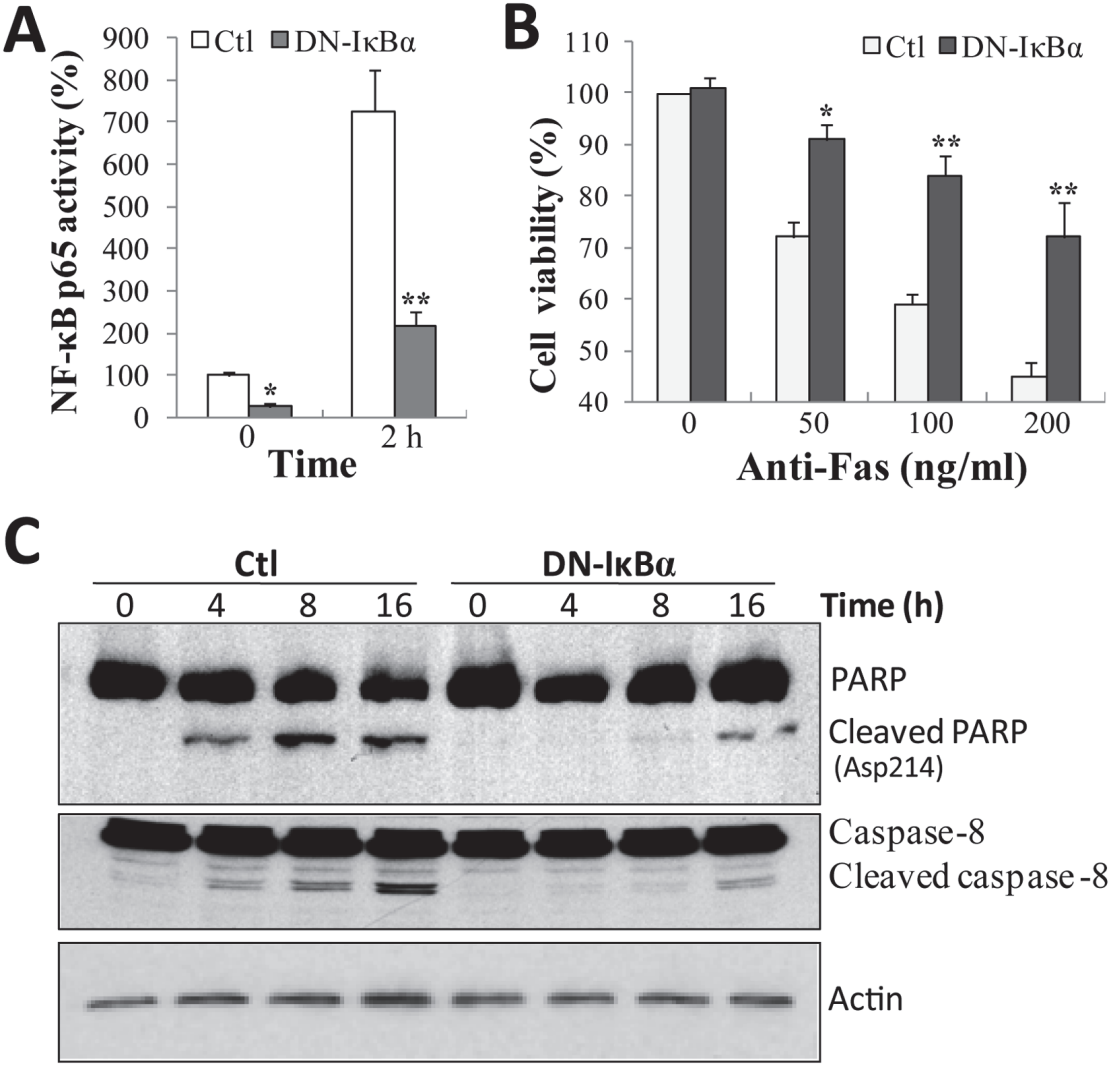
NF-κB inhibition protects neuroblastoma cells from Fas-induced apoptosis. (**A**) SH-EP1 cells transfected with control vector (Ctl) or DN-IκBα expression vector (DN-IκBα) were treated with anti-Fas antibody (100 ng/ml) for 2 h. NF-κB activation was analyzed using NF-κB p65 reporter assay. (**B**) Cells were treated with anti-Fas antibody of different concentrations for 1 d, and cell viability was examined using crystal violet staining. (**C**) Fas-treated cells were lyzed and Western blotting was performed with antibodies against cleaved PARP and caspase-8. Membranes were re-probed with β-actin as a loading control. Results are representative of at least three experiments. **P <0.01 and ***P <0.001, compared with control SH-EP1 cells.

To determine the role of NF-κB in Fas-induced cell death, SH-EP1 cells were transfected with DN-IκBα, a dominant negative form of IκBα (also named as IκBα-M) [21], which is a mutated IκBα at its two key phosphorylation sites (Ser32/36) preventing its phosphorylation and subsequent activation. As shown in Fig. 2A, in stable DN-IκBα-expressing SH-EP1 cells, the basal level of NF-κB activity was significantly attenuated, compared to control cells. When subjected to Fas stimulation, NF-κB activation in DN-IκBα cells was also remarkably inhibited (Fig. 2A). Taken together, these data suggest that DN-IκBα could efficiently block NF-κB activation under our experimental conditions.

Next, the effect of DN-IκBα on Fas-induced cell death in SH-EP1 cells was examined. To our surprise, DN-IκBα cells were more resistant to Fas-induced cell death than control cells (Fig. 2B). Similar results were obtained in several independent clones of DN-IκBα cells (data not shown). These data apparently demonstrate that the activation of NF-κB by Fas plays a pro-apoptotic role in SH-EP1 cells.

Meanwhile, the effect of DN-IκBα on cell apoptotic marker, PARP, was investigated (Fig. 2C). In line with the results of cell viability assessment, Fas-induced PRAP cleavage was strongly inhibited or much delayed in DN-IκBα cells. In addition, the effect of DN-IκBα on activation of caspase-8 was also assessed, because it is a well-known initiator caspase in Fas-induced apoptosis [3,5]. The results show that caspase-8 was clearly cleaved upon Fas treatment in control cells. However, in DN-IκBα cells, Fas-induced caspase-8 cleavage was greatly retarded. Therefore, the combined data reveal that NF-κB activity is required for both Fas-induced apoptosis and caspase-8 activation in SH-EP1 cells.

### NF-κB inhibition downregulates the expression of caspase-4

Considering that Fas/FasL expression is always regulated in Fas-induced cell death, we assessed whether the expression of Fas/FasL is affected by NF-κB in our system [28,29]. However, through Western blotting analysis, we did not find any significant difference for FasL protein expression in both control cells and DN-IκBα cells (S1 Fig.). As to Fas protein, because the protein level of Fas remained undetectable in both cells (data not shown), qPCR experiment was conducted. However, no obvious change could be detected in terms of *Fas* mRNA (S2 Fig.). Since FADD is the most important mediator for Fas killing [5–7], we also examined the effect of DN-IκBα on its expression. Unfortunately, no significant difference in FADD protein levels was observed between control cells and DN-IκBα cells (S1 Fig.). Together, these results suggest that other unknown mechanisms may be involved in NF-κB-mediated Fas killing.

**Fig 3:**
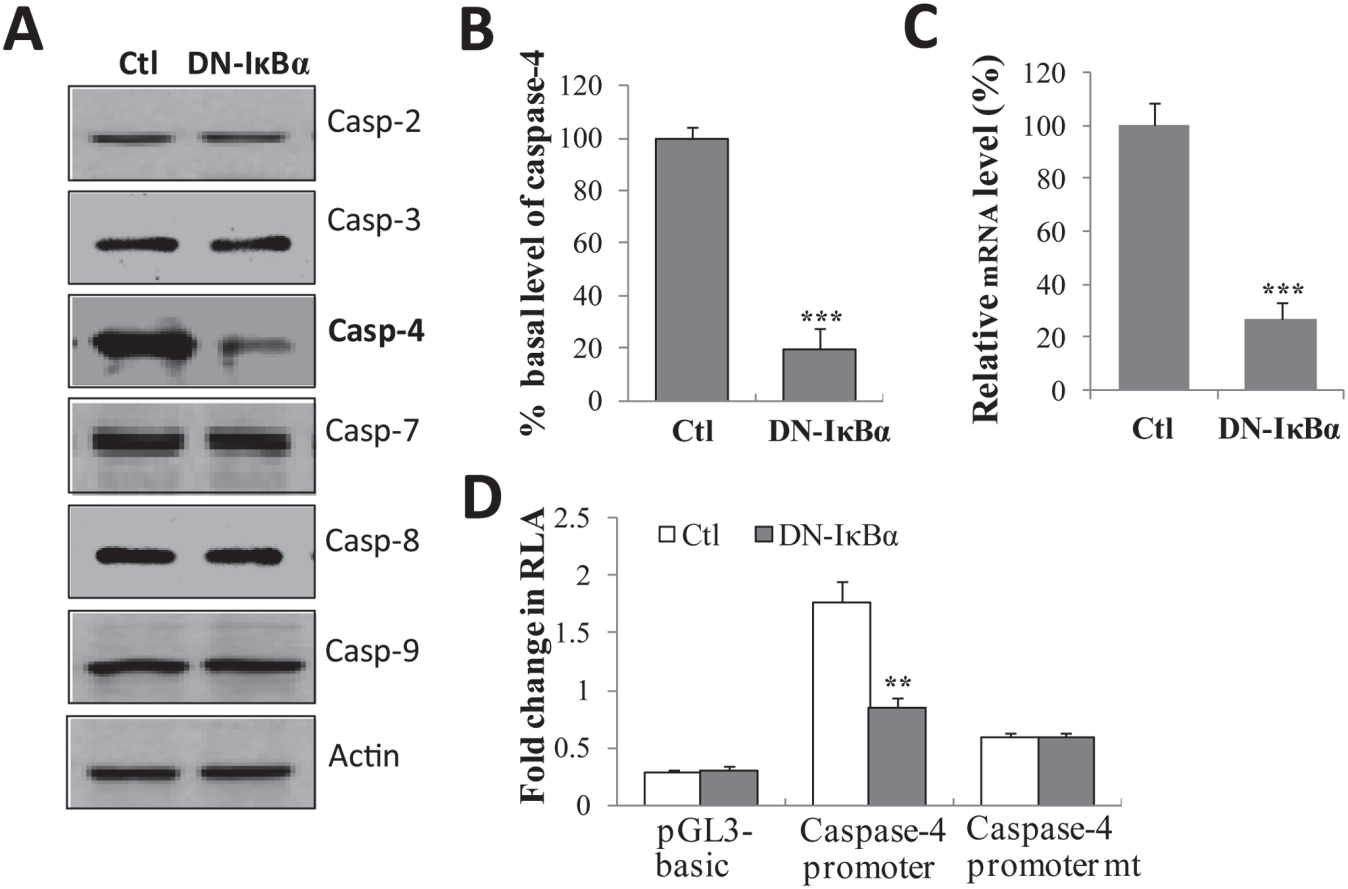
NF-κB inhibition down-regulates the expression of caspase-4. (**A**) SH-EP1 cells transfected with control vector (Ctl) or DN-IκBα expression vector (DN-IκBα) were lysed, and the expression levels of various caspases were detected using corresponding antibodies. (**B**) The expression level of caspase-4 was quantified. The caspase-4 level of DN-IκBα cells is expressed as a percentage of the level measured in control SH-EP1 cells. (**C**) The relative mRNA levels of caspase-4 in DN-IκBα cells were determined by qPCR. (**D**) The dual luciferase reporter assay was performed to evaluate the effect of DN-IκBα on caspase-4 promoter activity. DN-IκBα could significantly induce the activity of caspase-4 promoter, NF-κB binding site, but not NF-κB binding site mutant (mt). RLA represents relative luciferase activity. Data are representative of three independent experiments and shown as average ± s.e.m. (bars) values. **P <0.01 and ***P <0.001.

We further investigated whether various caspases are regulated by NF-κB signaling. The Western blotting result showed that there are negligible changes for caspase-2, -3, -7, -8 and-9 between control cells and DN-IκBα cells. In contrast, the protein level of caspase-4 was markedly downregulated in DN-IκBα cells, (Fig. 3A and 3B), reflecting that the constitutive level of NF-κB activity may be required for the expression of caspase-4.

Since caspase-4 contributes to Fas-induced apoptosis [32] and NF-κB regulates target genes mostly at the level of transcription, we hypothesized that caspase-4 might be regulated by NF-κB. Careful analysis of caspase-4 gene promoter revealed one potential NF-κB binding site (GGGAATCCCC) from-1,026 to-1,017 of upstream of caspase-4 open reading frame. If this binding site does play function, obviously the mRNA levels of caspase-4 should show difference between DN-IκBα and control SH-EP1 cells. As expected, results from qPCR experiment showed that the mRNA level of caspase-4 in DN-IκBα cells is significantly downregulated compared with that in control cells (Fig. 3C), demonstrating that the blockage of NF-κB activation reduces the transcription of caspase-4.

Next, we cloned human caspase-4 promoter region (bp −1030 to −74 upstream of the translation initiation site) into luciferase reporter vector pGL3-basic. Dual-luciferase reporter assay was performed to determine whether the expression of caspase-4 gene is NF-κB-dependent. As expected, in DN-IκBα cells, the activity of capsase-4 promoter is significantly suppressed, compared to control cells (Fig. 3D). However, when NF-κB binding site of caspase-4 promoter was mutated, the activities of capsase-4 promoter in control and DN-IκBα cells were both greatly attenuated (Fig. 3D). Taken together, these data indicate that NF-κB directly regulates the gene expression of caspase-4.

### NF-κB activation upregulates the expression of caspase-4

**Fig 4:**
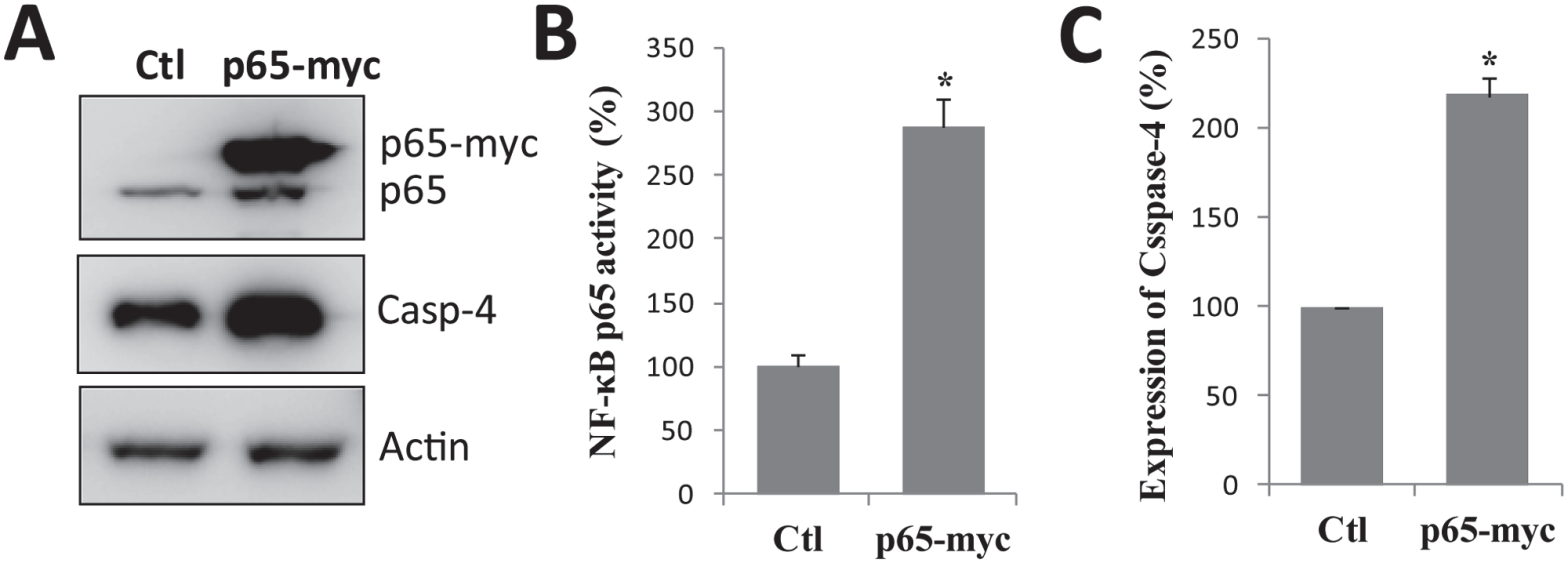
NF-κB p65-overexpression increases caspase-4 expression. (**A**) NF-κB p65 was transiently overexpressed in SH-EP1 cells, and the expression of p65 and caspase-4 were analyzed by Western blotting with the corresponding antibodies. (**B**) The expression level of caspase-4 protein was quantified. The caspase-4 level of p65-overexpressing cells is expressed as a percentage of that measured in control SH-EP1 cells. (**C**) The relative mRNA levels of caspase-4 in p65-overexpressing cells were determined by qPCR. Data are representative of three independent experiments and shown as average ± s.e.m. (bars) values. ***P <0.001.

In order to further confirm that caspase-4 expression is NF-κB-dependent, we determined whether constitutive activation of NF-κB upregulates caspase-4 in SH-EP1 cells. It was reported that overexpression of NF-κB p65 (*RelA*) could induce constitutive activation of NF-κB transcriptional activities in various cell lines [30,31]. Therefore, we transfected cells with NF-κB p65 (*RelA*) expression plasmid, and ectopic p65 was overexpressed with a level much higher than that of endogenous p65 (Fig. 4A). As expected, overexpression of p65 did lead to constitutive NF-κB activation (Fig. 4B). Meanwhile, caspase-4 expression was significantly upregulated in p65-overexprssing cells (Fig. 4C). To conclude, all these results further support our conclusion that the expression of caspase-4 is regulated by NF-κB. However, the fact that the Fas induces NF-κB activation but does not upregulate caspase-4 expression, suggests that Fas may act on other transcriptional factors to antagonize the induction of caspase-4 gene by NF-κB.

### Fas induces caspase-4 cleavage through NF-κB signaling

**Fig 5:**
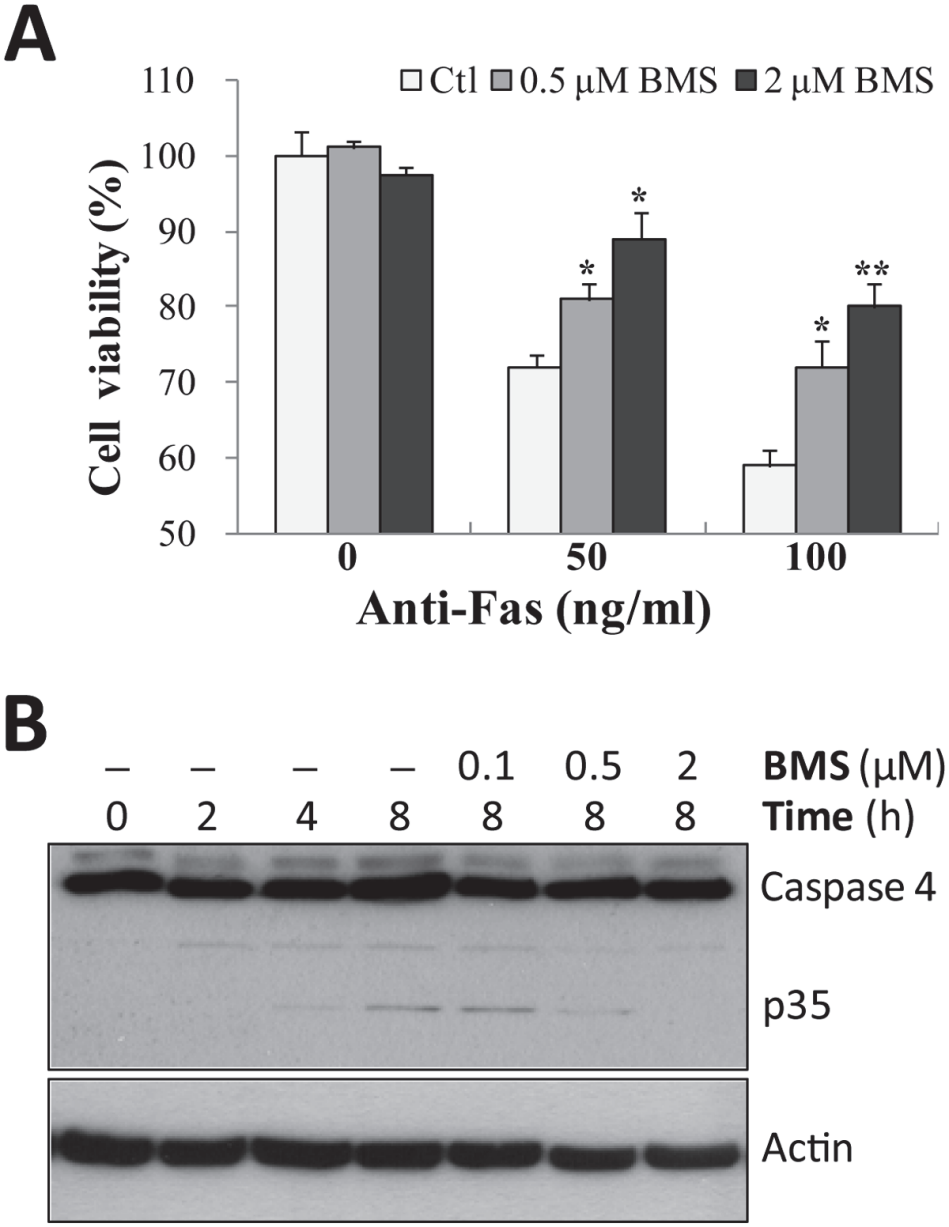
Fas induces caspase-4 cleavage through NF-κB signaling. (**A**) In the presence of IKK inhibitor BMS-345541 (BMS), SH-EP1 cells were treated with agonistic Fas antibody for 1 d, and cell viability was examined using crystal violet staining. (**B**) Western blotting was performed to detect the Fas-induced cleavage of caspase-4, in the presence or absence of BMS. Data are representative of three independent experiments and shown as average ± s.e.m. (bars) values. *P <0.05 and **P <0.01.

Since the expression level of caspase-4 protein seems not to be affected by Fas-induced activation of NF-κB (S1 Fig.), we hypothesized that the activity of caspase-4 might be affected by Fas via NF-κB. A specific inhibitor of IKK, BMS-345541 (BMS) was applied to SH-EP1 cells. As expected, BMS could significantly inhibit Fas-induced cell death in a dose-dependent way (Fig. 5A), again implying a pro-apoptotic role of NF-κB in Fas killing on SH-EP1 cells.

Further analysis with Western blotting confirmed our hypothesis that the activity of caspase-4 is affected by Fas, as shown by a significant increase of cleaved caspase-4 (active form of caspase-4 with 35 kDa) upon Fas stimulation (Fig. 5B). However, the presence of BMS at all concentrations could reverse Fas-induced increase in caspase-4 cleavage. Taken together, these results indicate that Fas can increase the activity of caspase-4 through NF-κB signaling.

### Caspase-4 is required for Fas-induced neuroblastoma cell apoptosis

**Fig 6:**
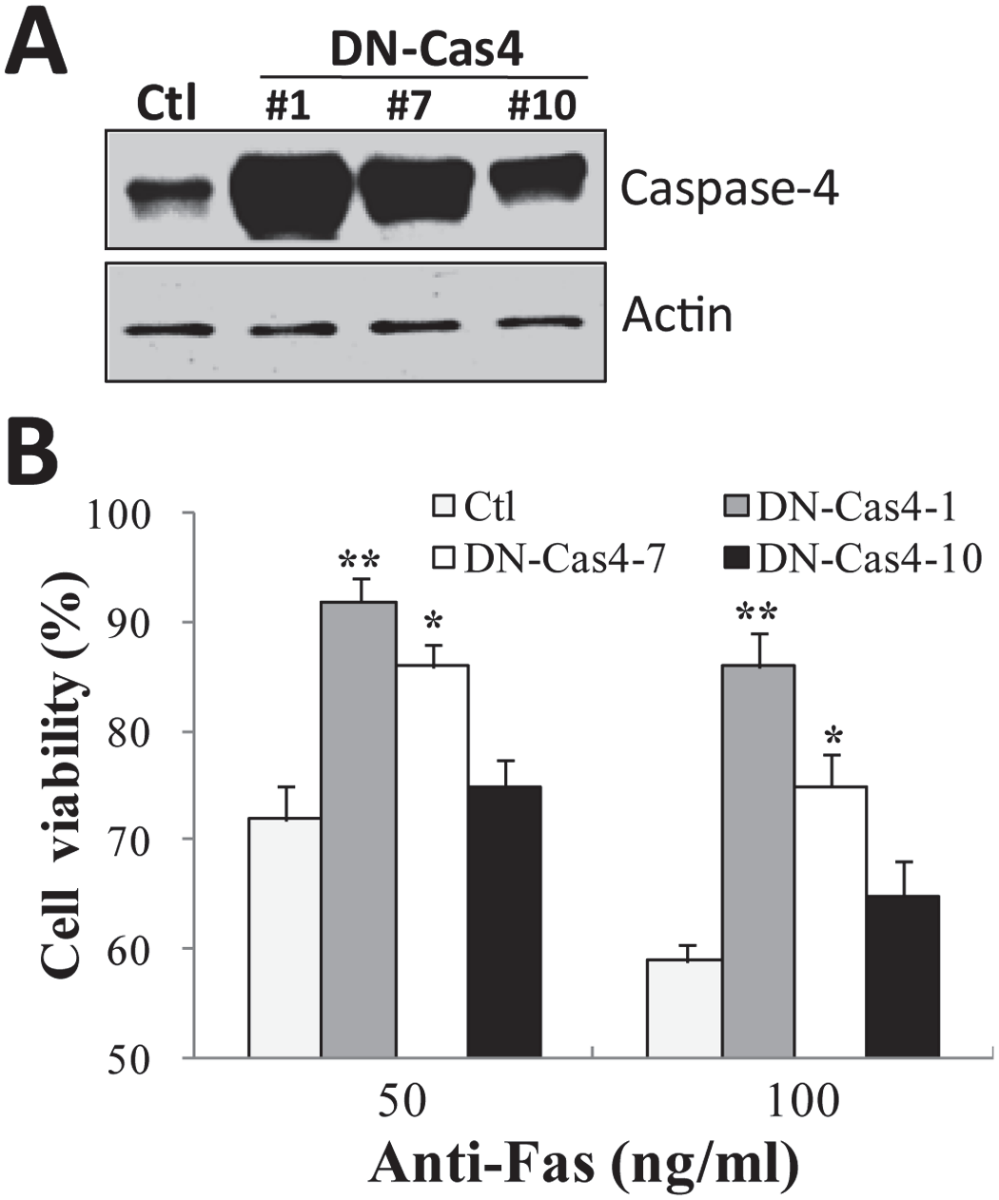
Caspase-4 is required for Fas-induced neuroblastoma cell apoptosis. (**A**) SH-EP1 cells transfected with control vector (Ctl) or DN-Cas4 expression vector (DN-Cas4) were subjected to Western blotting analysis with anti-caspase-4 antibody. #1, #7, and #10 represent selected single cell clones with different levels of overexpression of DN-Cas4.Membranes were re-probed with β-actin as a loading control. Results are representative of three experiments. (**B**) DN-Cas4 cells were treated with anti-Fas antibody of different concentrations for 1 d, and cell viability was examined using crystal violet staining. *P <0.05 and **P <0.01.

Previous study on caspase-4 contributing to Fas-induced apoptosis in some cells [32] strongly indicates that lower levels of caspase-4 protein may decrease the sensitivity of DN-IκBα cells to Fas killing. To test this hypothesis, SH-EP1 cells were transfected with dominant negative caspase-4 (DN-Cas4) expression vector [32], and several stable cell lines were selected (Fig. 6A). As expected, high expression level of DN-Cas4 in SH-EP1 cells reduced the cell death induced by Fas, compared to control vector-transfected cells (Fig. 6B). Meanwhile, the resistance degrees of cells to Fas killing appeared to be dependent on the expression levels of DN-Cas4, clearly indicating a pro-apoptotic role of caspase-4.

### Caspase-4 acts upstream of PARP and caspase-3 and downstream of caspase-8

**Fig 7:**
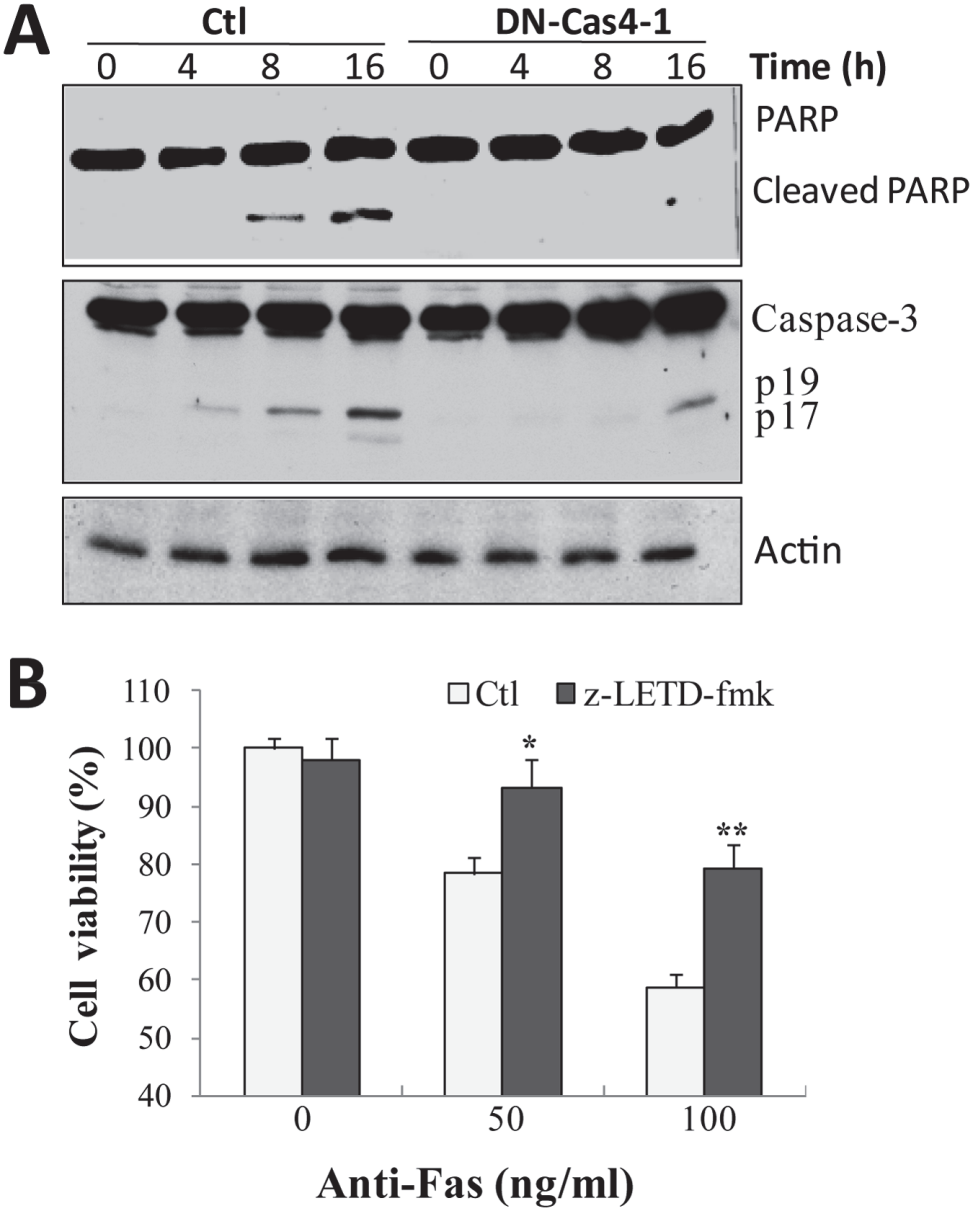
Caspase-4 acts upstream of PARP and caspase-3, but may play downstream of caspase-8. (**A**) SH-EP1 cells transfected with control vector (Ctl) or DN-Cas4 expression vector (DN-Cas4) were treated with Fas antibody (100 ng/ml) for 0, 4, 8, or 16 h, and Western blotting was performed with antibodies against cleaved PARP and caspase-3. β-actin was used as a loading control. Results are representative of at least three experiments. (**B**) In the presence or absence of caspase-8 inhibitor, z-LETD-fmk (30 μM), Fas-induced cell death was examined using crystal violet staining. *P <0.05 and **P <0.01.

Our results have pointed out that caspase-4 is required for Fas-induced cell death in SH-EP1 cells, but the action order of caspase-4 and other caspases in Fas killing is not clear. Thus, we examined the activation of PARP, caspase-8 and-3 by Fas stimulation in control cells and DN-Cas4 SH-EP1 cells. As shown in Fig. 7A, in DN-Cas4 cells, the cleavage of PARP and caspase-3 was almost completely inhibited or much delayed, when compared with control SH-EP1 cells. However, the cleavage of caspase-8 remained unchanged (data not shown), indicating that caspase-8 may act upstream of caspase-4.

Since caspase-8 is well known to play pro-apoptotic roles in Fas killing in other studies [3,4,6], we also examined the role of caspase-8 in our study. We introduced its specific inhibitor, z-LETD-fmk, into cell killing assay. The results showed that z-LETD-fmk at 30 μM drastically rescued Fas-induced apoptosis in SH-EP1 cells (Fig. 7B), indicating a pro-apoptotic role of caspase-8 in Fas killing, like caspase-4. Conclusively, these data indicate that caspase-4 may transduce Fas-induced death signals from caspase-8 to caspase-3 and PARP.

## Discussion

It is well known that Fas-induced apoptosis occurs in both differentiated neurons in adults and mitotic neurons in development, indicating an important role of Fas signaling in nervous system [8]. However, the detailed mechanism underlying Fas-induced apoptosis in nervous system or neuroblastoma cells has not been elucidated. In the present study, we demonstrated the mechanism responsible for the Fas-induced apoptosis in neuroblastoma cells. In brief, NF-κB mediates Fas-induced cell apoptosis via regulating caspase-4, which transduces death signal from caspase-8 to caspase-3 and PARP in neuroblastoma cells.

As a member of TNF family receptors, Fas can lead to the activation of NF-κB in many cell types. Although the activation of NF-κB has been considered to be part of the apoptotic induction, a lot of evidence suggests that NF-κB activation is an anti-apoptotic response in most circumstances [8,19,33]. For example, in leukemic eosinophil cells, activation of NF-κB by Fas confers resistance to Fas-induced apoptosis [33]. Similarly, the constitutive activity of NF-κB was also identified as a reason for the apoptosis-resistance against Fas in human ESCs [34]. However, there is still some evidence showing a dispensable role of NF-κB activation in Fas-induced apoptosis [17,35]. The dissociation of NF-κB from cell protective effects is based on two facts. One is that the inhibition of NF-κB has little effect on Fas-induced apoptosis [36], and the other is that the death still occurs in these cell types despite of the activation of NF-κB [17,35]. Interestingly, our data demonstrate a pro-apoptotic effect of NF-κB activation in Fas-induced apoptosis, distinctly different from previous findings. In the present study, agonistic anti-Fas antibody led to NF-κB p65 translocation to nucleus and NF-κB activity was greatly elevated. Notably, the activation of NF-κB was earlier than the apoptotic event. The activation of NF-κB peaked at 2 h post Fas treatment (determined by reporter assay), while the apoptosis occurred at 4 h (hallmarked by apoptotic marker PARP). These data indicate that NF-κB plays a role in Fas killing, which was validated by our following studies. In DN-IκBα cells resistant to NF-κB activation, Fas-induced cell death was significantly suppressed and apoptotic marker PARP also less cleaved. Taken together, our data clearly reveal NF-κB as an apoptotic mediator of Fas killing, rather than a pro-survival or dispensable one. In agreement with our conclusion, the results from two other studies also support a pro-apoptotic role of NF-κB activation in Fas killing in other cell types [24,25]. In gilioblatoma cells, NF-κB inhibition reduces the recruitment of FADD and caspase-8 and formation of the DISC upon stimulation of Fas. In endothelial cells, DN-IκBα protects cells from Fas-induced apoptosis and inhibits expression of caspase-8 inhibitor FLICE-inhibitory protein (c-FLIP). Collectively, it is possible that the role of NF-κB activation in Fas killing is cell type-dependent.

NF-κB activation mediating Fas killing on SH-EP1 cells rather than conferring cell protection suggests an alternative mechanism. The protective function of NF-κB has been extensively studied with many of its protective target molecules already identified, such as Bcl2, c-IAP、XIAP, and c-FLIP [3,37]. The pro-apoptotic function of NF-κB has also been studied [20,21,23] and the potential downstream death genes include p53 and c-myc, Fas/FasL, and Bax [3]. In this work, we utilized qPCR or Western blotting to assess whether these gene expression are regulated in DN-IκBα cells. Unfortunately, we did not detect any significant difference of these genes between control and DN-IκBα SH-EP1 cells (qPCR data for Bcl2, c-IAP、XIAP, c-FLIP, p53, and c-myc not shown). Interestingly, we found that caspase-4 is strongly downregulated in DN-IκBα cells, despite of no influence of Fas on the expression of caspase-4 gene in both cells apparently (S1 Fig.). More convincingly, caspase-4 promoter reporter assay further supported that NF-κB can directly transactivate caspase-4 gene expression. Together, it raised a question why Fas led to NF-κB activation, but no induction of caspase-4 gene was observed in our study. As one possible explanation, Fas may act on other transcriptional factors to antagonize the induction of caspase-4 gene by NF-κB. Another explanation is that NF-κB activity induced by Fas may not be sufficient to transactivate caspase-4 gene under our experimental conditions.

Next, we demonstrated that caspase-4 activity is required for Fas killing. When caspase-4 activity was blocked by its dominant negative form, cell death induced by Fas was almost completely abrogated in SH-EP1 cells. Consistently, the cleavages of apoptotic marker PARP and caspase-3 were also significantly blocked in caspase-4 DN cells. However, cleavage of caspase-8 remained unchanged in caspase-4 DN cells. On one hand, these data support the involvement of caspase-4 in apoptosis. On the other hand, they suggest that caspase-4 may function as a downstream effector of caspase-8, but acts upstream of caspase-3 and PARP in Fas-triggered caspase cascades. It is well known that caspase-8 is the initiator caspase within the DISC [6]. Its recruitment and activation by Fas/FasL complex is required for apoptosis. After activation, caspase-8 mediates proteolytic activation of effector caspase-3 and-7 and suffices for efficient apoptotic induction. Consistent with other previous finding, the pro-apoptotic effect of caspase-8 was also observed in our study, as shown by caspase-8-specific inhibitor greatly rescuing Fas killing. Taken together, caspase-4 may serve as an immediate caspase transducing death signal between initiator and effector caspases. Coincidently, Kamada et al. has shown that caspase-4 is required for Fas-induced apoptosis in HepG2 cells and could transmit the death signal from caspase-8 to caspase-3 proteases [32]. However, the details are still not clear about how caspase-4 transduces death signal between caspase-8 and caspase-3. It will be of great significance to further investigate the death signal transduction mechanism by caspase-4 in Fas killing.

Caspase-4 belongs to caspase-1 sub-family member, and mostly functions as a mediator or regulator of TNF-induced NF-κB signaling. For example, it interacts with TNF receptor-associated 6 and mediates lipopolysaccharide-induced NF-κB activation [37]. It is required for the induction of NF-κB activity in TNF-α signaling [38]. Therefore, its gene expression regulation is of great interest, especially regarding cell death. Frank et al. demonstrated that caspase-4 is a direct p53 target gene, but both p53 and NF-κB are required for full induction of caspase-4 gene in ionizing radiation-induced apoptosis in thymus cells [39]. Consistently, our results also demonstrated a crucial role of NF-κB in controlling caspase-4 gene expression. The difference is that caspase-4 acts as a NF-κB direct target gene in our study rather than a p53 target one. In our study, we also found that caspase-4 expression is NF-κB p65-dependent, as evidenced by increased caspase-4 expression in p65-overexpressing cells. Not uniquely, another NF-κB isoform, c-Rel, was also found to be associated with caspase-4 expression in EBV-transformed cells [40]. However, the authors demonstrated that c-Rel negatively modulates the expression of caspase-4. Collectively, NF-κB isoforms p65 and c-Rel play reverse roles in these two scenarios. Thus, the underlying mechanism for various roles of different NF-κB isoforms in regulating caspase-4 expression needs to be further addressed.

In summary, this study reveals a pro-apoptotic role of NF-κB signaling in Fas-induced apoptosis in neuroblastoma cells. In addition, caspase-4 was identified as a new target gene of NF-κB and a mediator of Fas-induced apoptosis. Caspase-4 may transduce apoptotic signaling from caspase-8 to caspase-3 and PARP. Considering Fas/FasL as an important defense system, it is expected to develop a new therapeutic strategy targeting caspase-4 in tumors, especially neuroblastoma cells in human.

## Materials and Methods

### Materials and cell culture

Human SH-EP1 neuroblastoma cells (ATCC CRL-2269) were provided generously by Dr. Goillot (Laboratoire d^’^Immunologie, Centre Leon Berard, Lyon, France) [41] and maintained in Dulbecco’s modified Eagle’s medium containing 10% fetal bovine serum, 100 units/ml penicillin, and 100 μg/ml streptomycin. Lipofectamine 2000 (Life Technologies Inc.) was used for transfections according to the manufacture’s procedures (Upstate). Agonistic anti-Fas antibody (CH-11) was bought from Medical and Biological Laboratories (Japan). Antibodies for human caspase-2, -3, -4, -7, -8, -9, and antibodies for cleaved PARP, caspase-3, -8, and-9 were from Cell Signaling Technology (Danvers, MA). Antibodies against Bax, Fas, FasL, FADD, and p65 were from Santa Cruz Biotechnology. Antibody against β-actin was purchased from Sigma-Aldrich (Germany). Caspase-8-specific inhibitor, z-LETD-fmk (Z-Leu-Glu(OMe)-Thr-Asp(OMe)-fmk), was purchased from Calbiochem. DN-IκBα, dominant-negative inhibitor of NF-κB and control vector was kept by our laboratory [21]. DN-Cas4, dominant negative inhibitor of caspase-4 was a gift from Dr. Kamada (Salk Institute, USA). All stably transfected cell clones were selected 800 μg/ml G418 (Life Technologies, Inc.) and maintained with 500 μg/ml G418. The complete protease inhibitor cocktails were purchased from Roche (Germany).

### Construction of plasmids

The caspase-4 promoter region (bp −1030 to −74 upstream of the translation initiation site) was amplified by PCR using human genomic DNA as a template with primers CAS4 (−1030): 5′-GGG GTA CCG GGC TGG GGG AAT CCC CCT T-3’ and CAS4 (−74): 5′-TCC CCC GGG TTC CTT ATT GCA AAG GGC GGA-3′. A mutant (GG**A G**AT **T**CC C) of candidate NF-κB binding site (GGG AAT CCC C) was generated with same protocol, except using a different forward primer (5′-GGG GTA CCG GGC TGG GGG AAT CCC CCT T-3′) with point mutation on the NF-κB binding site. The PCR products were then cloned into *Xho* I/*Kpn* I sites of pGL3-Basic (Promega), a luciferase reporter vector.

A wild-type p65 (*RelA*) was amplified by PCR using human cDNA as a template with primers hRelA-F: 5′- CGG GAT CCG ACG AAC TGT TCC CCC TCA TC-3′ and hRelA-R: 5′-ACC GCT CGA GTT AGG AGC TGA TCT GAC TCA GCA G-3′. The PCR products were cloned into *Bam*H I*/Xho* I sites of pXJ40-myc.

### Luciferase reporter assay

Cell lysates were subjected to a dual-luciferase reporter assay according to the instructions of the manufacturer (Promega). Briefly, an appropriate amount of the caspase-4 promoter luciferase reporters, together with Renilla luciferase plasmids, which served as an internal control, were co-transfected into cells. 48 h later, cell lysates were subjected to a dual-luciferase reporter assay. The luciferase activities for the promoter reporters were detected by GloMax 96 Microplate Luminometer (Promega) and normalized to activities of Renilla luciferase. The data represented at least three independent experiments.

### Cell viability assessment

Fas-induced cell death was assessed using crystal violet staining as described previously [21]. In brief, 2×10^4^ cells were plated in 96-well plates in a triplicate manner. Medium was changed and supplemented with Fas antibody on the next day for up to 24 h. The plate was stained with 0.5% crystal violet in 20% methanol for 20 min at room temperature and then washed with tap water. Stains were dissolved with 20% acidic acid, and measured at wavelength 570 nm with Tecan reader (Männedorf, Switzerland).

### Immunocytochemistry

Nuclear translocation of NF-κB p65 was analyzed by immunocytochemistry as described previously [21]. Briefly, SH-EP1 cells were treated with agonistic anti-Fas antibody for 1 h and fixed with 4% paraformaldehyde. The cells were incubated with a rabbit polyclonal antibodies against NF-κB p65 (Santa Cruz Biotechnology, USA) for 1 h followed by incubation with FITC-labeled goat anti-rabbit IgG for 1 h. Finally, cells were examined under a fluorescence microscope (Carl Zeiss, USA).

### Real-time PCR

Total RNA was extracted using Trizol reagent following the manufacturer’s instructions (Invitrogen), and reversely transcribed to cDNAs using SuperScript II reverse transcriptase (Invitrogen). Quantification of mRNA levels was measured by using real-time PCR system (ABI Prism7500, Applied Biosystems) and SYBR Green qPCR Master Mix (KAPA Biosystems). Gene-specific primers used for real-time PCR were as follows: 5′-TGC ATC ATG ATG GCC AAT TC-3′ and 5′-GCA GTT TAT TTC CAC TTC TAA G-3′ for Fas, 5′-TTG CTT TCT GCT CTT CAA CG-3′ and 5′-GTG TGA TGA AGA TAG AGC CCA TT-3′ for caspase-4, 5′-AGG GCT GCT GCT TTT AAC TCT GGT-3′ and 5′-CCC CAC TTG ATT TTG GAG GGA-3′ for GAPDH. The target mRNA level of control cells normalized to the level of GAPDH mRNA, was set to 1.

### Western blotting

Cells were washed twice with cold PBS, and then extracted with lysis buffer (20 mM Tris-HCl pH 7.5, 150 mM NaCl, 1 mM EDTA, 1 mM EGTA, 1% Triton X-100, 1 mM PMSF and Roche’s complete protease inhibitors) and centrifuged at 14,000 g for 20 min at 4°C. The protein concentration of the supernatants was determined using a Protein Assay Kit II (Bio-Rad). For Western blotting, samples were separated by electrophoresis on 10%**–**18% SDS**-**PAGE, and transferred onto PVDF membranes (Millipore Corp.). After blocking with PBST (PBS with 0.1% Tween-20) containing 5% skim milk, the membranes were incubated with primary antibodies. They were further incubated with horseradish peroxidase (HRP)-conjugated secondary antibodies and developed using Millipore’s chemiluminescence substrate. To determine the equivalence of protein amounts loaded among different samples, the developed membranes were stripped with a buffer consisting of 62.5 mM Tris-HCl (pH 6.7), 2% SDS and 100 mM 2-mercaptoethanol for 1 h, followed by incubation with reference antibody, such as anti-β-actin for further blotting. In some cases, immunoblots were quantified by measuring the immunoreactive protein band density with software ImageJ 1.48 (NIH, USA).

### NF-κB reporter assay

According to the manufacturer’s instructions, NF-κB TransAM Assay kits (Active Motif, CA) were used to detect DNA binding activity of p65 containing p50/p65 heterodimers [42]. Briefly, 10 μg of nuclear extracts of cells was incubated in oligonucleotide-coated 96-wells plate for 1 h. After washing, the bound complexes were incubated with antibodies against NF-κB p65 for 1 h. The plates were then incubated with HRP-conjugated secondary antibodies for another 1 h. Finally, the developing solution was applied for 10 min of incubation, followed by measurement of absorbance at 450 nm.

### Statistical analysis

Data were expressed as means ± s.e.m. values. The group means were compared by analysis of variance, and the significance of differences was determined by post hoc testing using Bonferroni’s method. Differences were considered significant at *P* <0.05.

## Supporting Information

S1 Fig Fas does not affect caspase-4 expression in appearance. SH-EP1 cells transfected with control vector (Ctl) or DN-IκBα expression vector (DN-IκBα) were treated with anti-Fas antibody (100 ng/ml) for a time course as indicated. Cells were lysed and Western blotting analysis was carried out using corresponding antibodies. Data are representative of three independent experiments.
(TIF)

S2 Fig Activation of NF-κB by Fas does not induce Fas expression. SH-EP1 cells transfected with control vector (Ctl) or DN-IκBα expression vector (DN-IκBα) were treated with anti-Fas antibody (100 ng/ml) for 1d. The relative mRNA levels of caspase-4 in DN-IκBα cells were determined by qPCR. Data are representative of three independent experiments and shown as average ± s.e.m.
(TIF)
