# Supplementary figures and images for: NF-κB Regulates Caspase-4 Expression and Sensitizes Neuroblastoma Cells to Fas-Induced Apoptosis
Source: PLoS One. 2015 Feb 19;10(2):e0117953. doi: 10.1371/journal.pone.0117953 (PMC4335045; doi:10.1371/journal.pone.0117953)

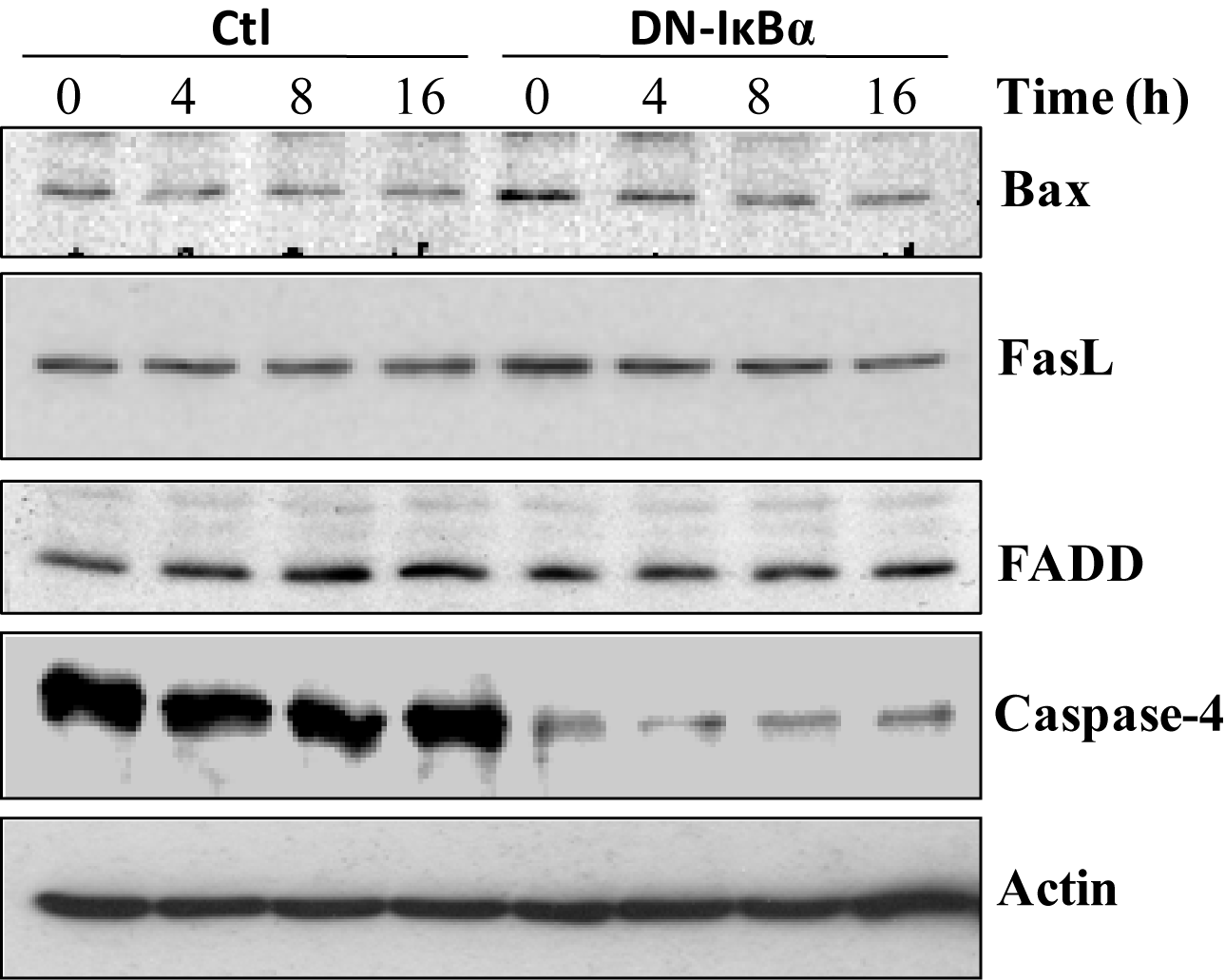

Supplement: S1 Fig — SH-EP1 cells transfected with control vector (Ctl) or DN-IκBα expression vector (DN-IκBα) were treated with anti-Fas antibody (100 ng/ml) for a time course as indicated. Cells were lysed and Western blotting analysis was carried out using corresponding antibodies. Data are representative of three independent experiments. (TIF) [file pone.0117953.s001.tif]

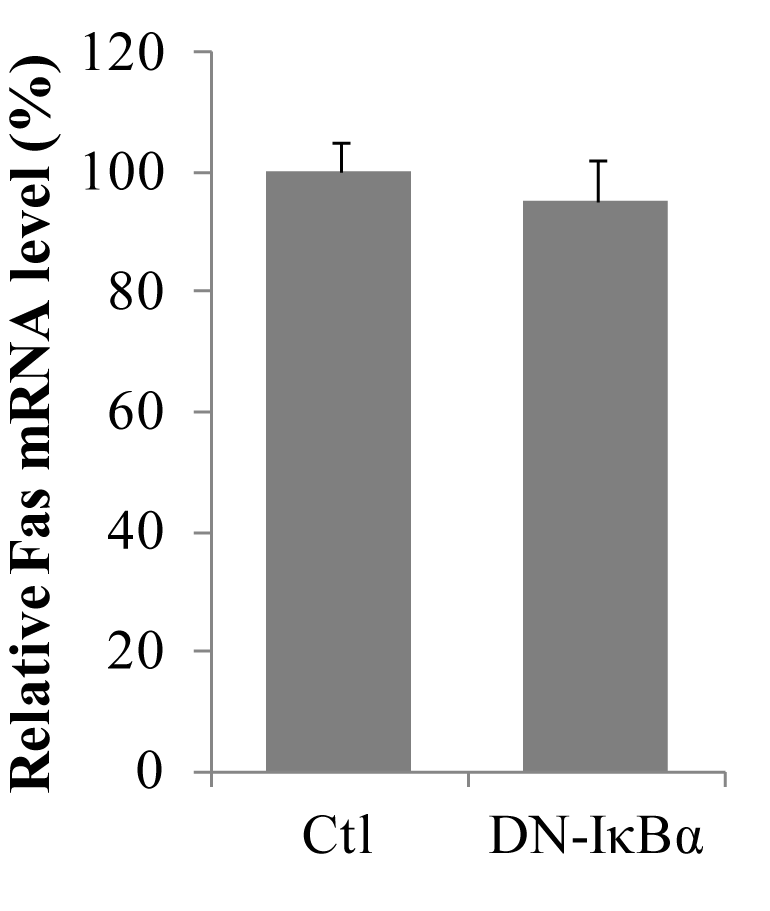

Supplement: S2 Fig — SH-EP1 cells transfected with control vector (Ctl) or DN-IκBα expression vector (DN-IκBα) were treated with anti-Fas antibody (100 ng/ml) for 1d. The relative mRNA levels of caspase-4 in DN-IκBα cells were determined by qPCR. Data are representative of three independent experiments and shown as average ± s.e.m. (TIF) [file pone.0117953.s002.tif]
